# New Rat Model Mimicking Sacrocolpopexy for POP Treatment and Biomaterials Testing via Unilateral Presacral Suspension

**DOI:** 10.1007/s00192-024-06019-4

**Published:** 2025-01-07

**Authors:** Chenxi Lu, Jun Zhou, Qingyu Kong, Lulu Wang, Wei Ni, Zhen Xiao

**Affiliations:** 1 Department of Obstetrics and Gynecology, First Affiliated Hospital of Dalian Medical University, 222 Zhongshan Road, Dalian, China; 2 Department of Obstetrics and Gynecology, Fengxian District Central Hospital, Shanghai, China; 3 Department of Gynecology, The People’s Hospital of Yingkou, Yingkou, China; 4 The People’s Hospital of Naqu, Naqu, China

**Keywords:** Pelvic organ prolapse, Rats, Animal model, Surgery, Materials, Biomaterials

## Abstract

**Introduction and Hypothesis:**

Pelvic organ prolapse (POP) impacts women’s health and quality of life. Post-surgery complications can be severe. This study uses rat models to replicate sacrocolpopexy and test materials for pelvic support, verifying the 4-week postoperative mortality rate, the mechanical properties of the mesh tissue, and the collagen content.

**Methods:**

Twenty-one 12-week-old female Wistar rats were used. Eighteen rats were subjected to POP induction by cervical suction and constant traction. One week after prolapse modeling, 18 prolapsed rats underwent unilateral presacral suspension (UPS) surgery with polycaprolactone (PCL) scaffolds, decellularized porcine small intestinal submucosa (SIS) scaffolds, or polypropylene (PP) meshes (*n* = 6 each). UPS rats were compared with normal rats (*n* = 3). After 4 weeks, conditions and mortality were recorded. The rats were then euthanized for biomechanical testing and collagen analysis. Ultimate load (N) was defined as the highest load before the failure of the target sample.

**Results:**

The UPS procedure requires 42.9 ± 4.5 min with no complications or deaths over 4 weeks. SIS was the stiffest mesh (14.53 ± 0.86 N), followed by PP (8.43 ± 0.40 N), and PCL was the least stiff (0.66 ± 0.05 N). After 4 weeks, the ultimate load of the PCL complex increased to 1.71 ± 0.41 N (*p* = 0.0120), but showed no significant difference from parametrial fascia (1.25 ± 0.85 N) and uterosacral ligament (0.66 ± 0.41 N). The ultimate load of the SIS complex decreased to 5.99 ± 0.37 N, still higher than native tissue. The PP complex’s ultimate load (10.02 ± 1.80 N) showed no significant difference from PP alone. The collagen ratio of the PCL complex (48.11 ± 9.88%) was closest to that of the uterosacral ligament (36.66 ± 11.64%), whereas SIS and PP complexes had significantly higher collagen ratios than USL.

**Conclusions:**

Unilateral presacral suspension mimics classical surgery for human POP in rats. First, this procedure can investigate the mechanical properties of pelvic floor tissues at the cellular level after correcting POP. Second, it can be used to validate new materials for the surgical treatment of POP, including but not limited to foreign body reactions with surrounding tissues, absorption time, etc. Third, it can be used to study the biological mechanisms of mesh exposure.

## Introduction

Pelvic floor dysfunction encompasses a range of interrelated clinical conditions, including pelvic organ prolapse (POP), urinary incontinence, fecal incontinence, urinary dysfunction, defecatory dysfunction, and sexual dysfunction [1, 2]. Beyond the physical symptoms, these conditions have a significant emotional impact, leading to psychosocial distress such as social isolation, anxiety, and depression [3]. Vaginal childbirth is the greatest independent risk factor for the development of POP [4]. Pelvic floor dysfunction affects nearly one-third of premenopausal women and almost half of postmenopausal women [5]. The global incidence of POP in 2019 was estimated to include 13 million new cases [6].

Surgical options for POP treatment include sacrocolpopexy, uterosacral ligament (USL) suspension, sacrospinous ligament fixation, native-tissue repair, and vaginal surgery [7–9]. Various synthetic meshes have been developed to treat POP, with polypropylene (PP) mesh being widely used [10]. However, these meshes come with associated risks of complications such as pain, exposure, and infection [11]. The US Food and Drug Administration (FDA) has reclassified synthetic mesh used for pelvic repair to the most stringent level of review [12].

Currently, the experimental animals widely used in POP research include rats, mice, and sheep. Pigs, rabbits, and nonhuman primates are used less frequently. These experimental animals have their respective advantages for different research purposes [13]. Nonhuman primates, because of their highly similar birthing process, reproductive cycles, estrous cycles, and pelvic floor anatomy to humans, were once considered the “gold standard” experimental animals for POP research [14, 15]. Ma et al. constructed bioengineered grafts by seeding human umbilical mesenchymal stem cells onto biological scaffolds to reconstruct damaged vaginas in rhesus monkeys [16]. Shapiro et al. implanted two commonly used PP single-incision slings into sheep vaginas to compare their biochemical and mechanical properties in host tissues [17]. Large animals are excellent models for POP research, but, owing to experimental conditions, researchers, research budgets, and the “3Rs” principle, small animals can be a viable alternative for most experiments.

Rodent models are commonly used in the study of POP in animals [18–21]. They have a pelvic floor structure similar to humans and are easy to obtain and manipulate [22, 23]. Various studies have been conducted on rats or mice using different materials (PP, hydrogel, silk fibroin, or polycaprolactone [PCL]) and methods (implanting into the interfascial space on the back, or securing the vaginal vault to the USL) to treat POP [24–27]. The structure and vascular richness of the skin fascia and pelvic fascia differ. To evaluate the mesh used for treating POP, it is better to place the mesh within the pelvic fascia for periodic observation and assessment.

In this study, it is a novel method to simulate clinical pelvic floor repair surgery sacrocolpopexy on rats. Building on our previous study [21], we conducted unilateral presacral suspension (UPS) on prolapsed rats, implanting different materials beneath the posterior peritoneum, to verify the 4-week postoperative mortality rate of the UPS, the mechanical properties of the mesh tissue, and the collagen content.

## Materials and Methods

### Animal Ethics and Animal Welfare

This study followed the guidelines of the Institutional Animal Care and Use Committee (IACUC) and obtained approval for the animal procedures before commencement. Sterile surgical techniques were required, as referenced in the Guide for the Care and Use of Laboratory Animals and the Federal Animal Welfare Regulations. The study was approved by the Institutional Animal Care and Use Committee of Dalian Medical University (No.AEE23058). Twelve-week-old nulliparous female Wistar rats were acquired (*n* = 21). After prolapse modeling [21], the prolapsed rats (*n* = 18) were housed in an animal room with standard humidity (45−50%), temperature (22 ± 2)°C, and a 12-h light cycle (12 h daylight/12 h dark), with free access to food and water.

### Mesh

Three prolapse meshes were used: PP mesh (*n* = 6), PCL scaffolds (*n* = 6), and decellularized porcine (SIS) scaffolds (*n* = 6). PP mesh is non-absorbable, whereas PCL and SIS scaffolds are absorbable. Before the UPS, the three meshes were soaked in 75% alcohol for 30 min, rinsed with PBS three times (each rinse lasting 1 min), and then exposed to UV light for 30 min. Uniaxial tensile testing was conducted on the three meshes, each cut into rectangular pieces measuring 20 mm in length and 2 mm in width.

### The Procedure of Unilateral Presacral Suspension

#### Prolapsed Rat Model

**Fig. 1:**
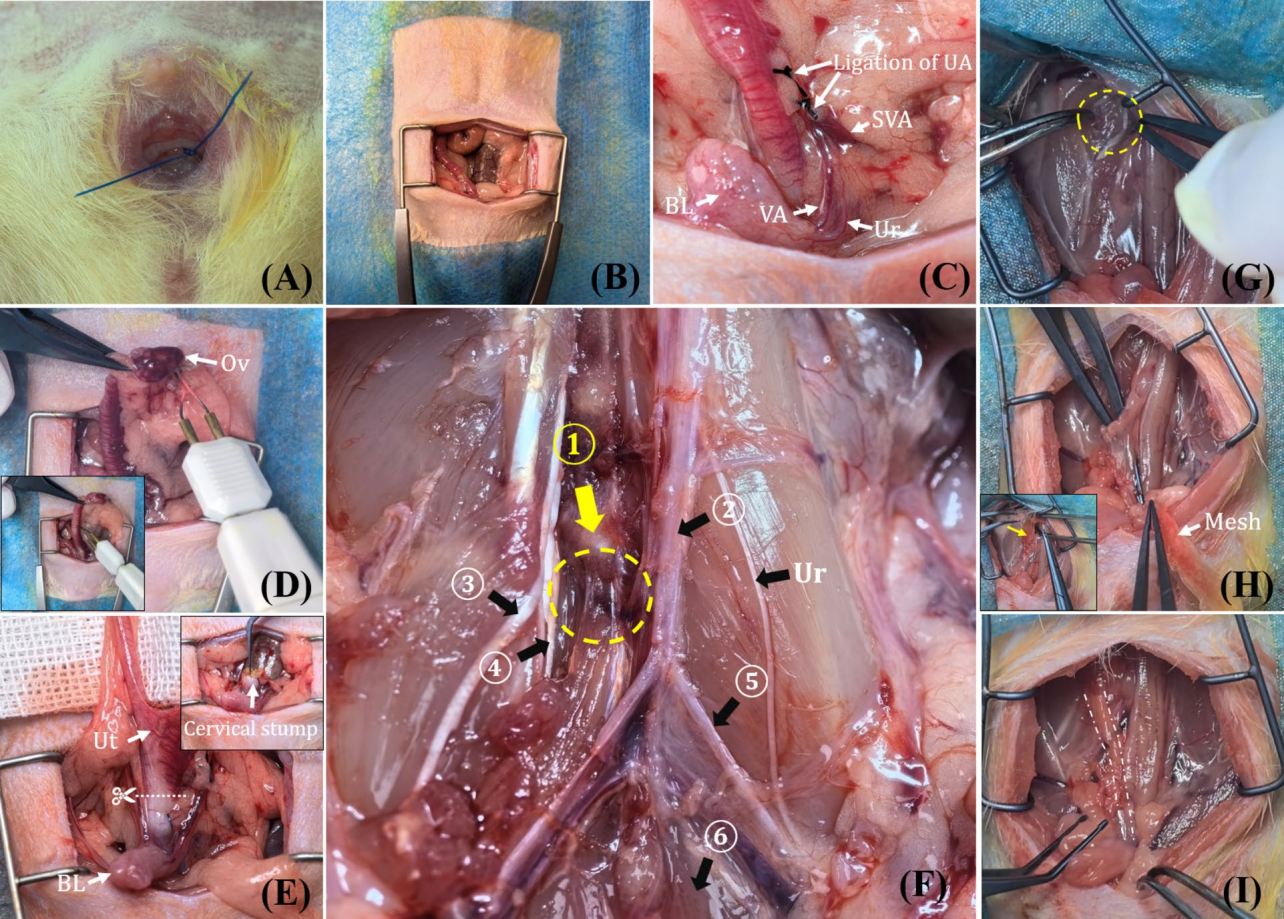
The procedure of unilateral presacral suspension (UPS). **A** The cervix of the prolapsed rat is exposed at the vaginal opening, with sutures from the prolapse modeling left in place to facilitate the evaluation of postoperative prolapse repair. **B** Exposing the abdominal cavity. **C** The anatomical relationship of the uterine artery (UA), the vesical artery (VA), the superior vesical artery (SVA) and the ureter (Ur). **D** Dissecting the bicornuate uterus and ovaries. **E** Subtotal hysterectomy. **F** Pelvic anatomy of the rat after removal of the posterior peritoneum and part of the presacral muscle group. The *yellow dotted area* indicates a strong prevertebral ligament, which is the site for mesh fixation. *1* First and second sacral vertebrae space; *2* abdominal aorta; *3* sixth lumbar nerve; *4* pudendal nerve; *5* common iliac artery; *6* sacrococcygeus ventralis medialis. **G** Confirming the fixation site (*yellow dotted area*). **H** Using angled forceps to create a mesh tunnel. **I** The status of mesh fixation and posterior peritoneum closed. The *white dotted area* indicates the position of the mesh.*Ov* ovary, *Ut* uterus, *BL* bladder

Based on our previously developed prolapsed rat model [21], a special vacuum suction device suitable for the rat's vagina was completely inserted into the rat's vagina. The rat's cervix and part of the fornix tissue were pulled outside the vaginal opening by negative pressure. The traction criterion was that the cervix was pulled 5 mm from the external vaginal introitus. The cervix was sutured with 3–0 Prolene sutures in a cross pattern, leaving a 2-cm-long thread for subsequent traction. Traction was performed nine times daily, each time for 2 min, with a 1-min rest interval, for 7 days. Then there were 7 days of recovery before UPS. The prolapsed cervix was exposed outside the introitus with sutures from the prolapse modeling left in place to facilitate the evaluation of postoperative prolapse repair (Fig. 1A).

#### Anesthesia and Disinfection

Before anesthesia, the rats were fasted for 12 h to minimize the impact of intestinal contents during surgery. Pentobarbital sodium (50 mg/kg) was administered via intraperitoneal injection to anesthetize the rats. After anesthesia, streptomycin (28 mg/kg) was injected into the inner thigh to prevent infection. The anesthetized rats were then placed on a constant temperature heating pad. The abdominal hair was removed, and the vagina and cervix were disinfected with iodophor.

#### Exposing the Abdominal Cavity

The abdominal hair was removed. After disinfection and draping, a 30-mm longitudinal incision was made along the midline of the abdomen, 15 mm above the urethral opening. The muscle groups were then separated, and a retractor was used to expose the abdominal cavity (Fig. 1B).

#### Ligation of the Bilateral Uterine Arteries

The superior vesical artery originates from the internal iliac artery, branching cranially to form the uterine artery and caudally to form the vesical artery [28]. Simultaneously, the lower segment of the ureter passes through the bifurcation of the superior vesical artery, entering the bladder along the outside of the vesical artery. Therefore, when ligating the uterine artery, it is crucial to avoid ligating other important blood vessels or the ureter (Fig. 1C).

#### Dissecting the Bicornuate Uterus and Ovaries

After ligating the uterine arteries on both sides, the ovaries and uterus could be freed. By grasping the lower edge of the ovary and the ovarian uterine artery, the electrocautery hemostasis pen was applied along the lower edge of the angled forceps to free the ovaries and uterus (Fig. 1D).

#### Subtotal Hysterectomy

The uterus was cut at the upper edge of the cervix, and the cervical stump was sutured with 8-0 non-absorbable silk. One end of the mesh was then fixed to the dorsal side of the cervical stump with 8-0 non-absorbable silk (Fig. 1E). Preserving the cervix allowed for the retention of the 3-0 Prolene sutures on the cervix, facilitating postoperative observation of the cervical suspension status.

#### Confirming the Anterior Vertebral Ligaments

Figure 1F illustrates the rat pelvic structure post-euthanasia, following the separation of the pelvic floor muscles. The space between the first and second sacral vertebrae is located to the right of the confluence of the abdominal aorta and the common iliac artery. The anterior vertebral ligaments, which are exposed, are robust enough to endure the force of cervical suspension.

#### Mesh Tunnel and Fixation

The retroperitoneum was opened to the right of the confluence of the abdominal aorta and the common iliac artery, exposing the anterior vertebral ligaments (Fig. 1G). Angled forceps were inserted into the retroperitoneum and exited below the cervical stump. These forceps grasped the other end of the mesh, positioning it entirely beneath the posterior peritoneum (Fig. 1H). The mesh was then pulled upward and secured to the anterior vertebral ligaments using 8-0 non-absorbable silk. To prevent the mesh from being exposed to the abdominal cavity and eroding organs such as the bladder and intestines, the posterior peritoneum was sutured (Fig. 1I). The abdominal cavity was closed, the skin was sutured and disinfected, and the rats were transferred to a constant temperature box for 24-h observation.

### Specimen Collection and Preservation

**Fig. 2:**
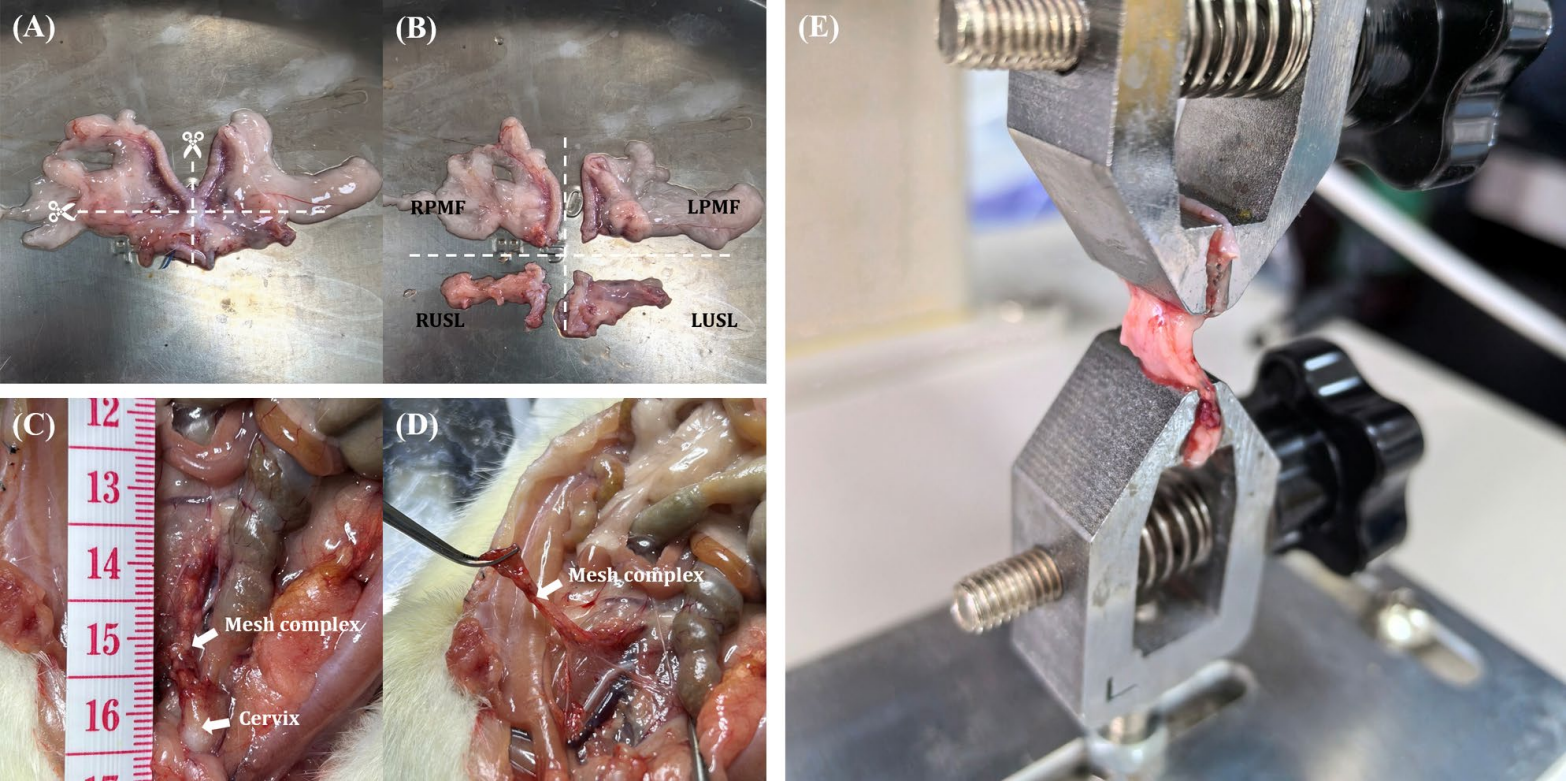
Biomechanical testing. **A** The intact uterus and its surrounding supporting tissues are flattened on the tray. The *horizontal dashed line* indicates the cut along the upper border of the cervix. The *vertical dashed line* indicates the cut along the midline of the cervix. **B** Four parts after cutting, right parametrial fascia (RPMF), left parametrial fascia (LPMF), right uterosacral ligament (RUSL), and left uterosacral ligament (LUSL). **C** Pelvic anatomy observed after opening the abdominal cavity. **D** Ligament-like structure 4 weeks after unilateral presacral suspension. **E** The PMF is secured on a mechanical testing machine. *RPMF* right parametrial fascia, *LPMF* left parametrial fascia, *RUSL*, right uterosacral ligament *LUSL* left uterosacral ligament

The parametrial fascia (PMF) and the USLs were collected from normal rats (*n* = 3). The uterus and its surrounding supportive tissues were completely removed and laid flat on the operating table. A horizontal incision was made along the upper edge of the cervix. The part connected to the uterine body was identified as the PMF, and the part connected to the cervix was identified as the USL (Fig. 2A, B) [22]. Four weeks after the UPS, the mesh complexes were collected from the prolapsed rats (*n* = 18; Fig. 2C, D).

### Mechanical Testing

The specimens tested on the uniaxial testing machine included three different meshes (PCL, SIS, and PP), PMF, USL, and mesh complexes (PCL complex, SIS complex, and PP complex). PMF and USL served as controls to compare the strength of the mesh complexes 4 weeks after UPS. Each specimen was mounted on the mechanical testing machine (Fig. 2E). Before each test, the specimen was preloaded to 0.05 N and then preconditioned for ten cycles at an extension rate of 25 mm/min within an elongation range of 0 to 2 mm, corresponding to cycling in the elastic region of the load-elongation curve. After preconditioning, a uniaxial failure test was immediately performed at the same extension rate. The load-elongation curve and failure mode were recorded from data points collected every 0.02 s using the provided software, then imported into Excel for analysis. The ultimate load (N) at failure was defined as the highest load before the failure of the target mesh tissue.

### Masson’s Trichrome Staining and Collagen Ratio Analysis

Masson’s trichrome staining was performed according to the manufacturer’s instructions. The slices were then observed and photographed using an optical microscope. Using the image-processing software ImageJ, four square areas around the mesh were randomly selected from the mesh complex slices, whereas the parts close to the cervix were selected from the USL slices. Both were analyzed on 10 × pathology slides to measure the collagen ratio.

### Statistical Analysis

Statistical analyses and graphical representations were performed using GraphPad Prism 9 (GraphPad Software, USA). Data are presented as mean ± standard deviation. A two-tailed Student’s *t* test was used to assess differences between the two groups. Differences among groups in ultimate load and collagen ratio were evaluated using a one-way analysis of variance. Statistical significance was defined as (*p* < 0.05).

## Results

### Unilateral Presacral Suspension

The average time of UPS was 42.9 ± 4.5 min. All the prolapsed cervixes changed from visible to invisible. None of the rats exhibited intraoperative complications during UPS. None of the rats died during the 4 weeks of observation.

### Mechanical Testing

**Fig. 3:**
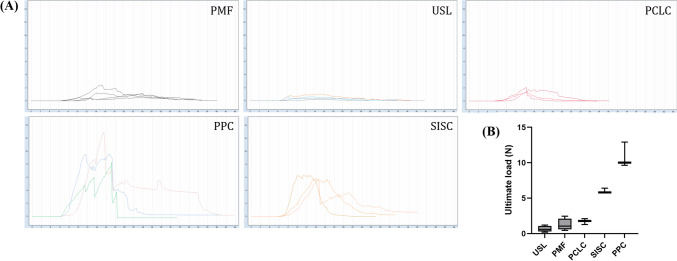
The result of mechanical testing. **A** Uniaxial testing curves of parametrial fascia (PMF), uterosacral ligament (USL), polycaprolactone scaffolds complex (PCLC), polypropylene mesh complex (PPC), and small intestinal submucosa scaffolds complex (SISC) generated following a load to failure. **B** Statistical analysis results of ultimate load 4 weeks after unilateral presacral suspension. Data are presented as mean ± standard deviation

Four weeks after the UPS, ligament-like tissue was observed, forming a new complex with the mesh, newly formed collagen fibers, and surrounding supportive connective tissue (Fig. 2C). As shown in Table 1, SIS was the stiffest mesh (ultimate load 14.53 ± 0.86 N), followed by PP (ultimate load 8.43 ± 0.40 N), and PCL was the least stiff (ultimate load 0.66 ± 0.05 N). The ultimate load of the PCL complex (1.71 ± 0.41 N) was statistically significant compared with PCL alone (*p* = 0.0120), but not statistically significant compared with PMF (1.25 ± 0.85 N) and USL (0.66 ± 0.41 N; *p* = 0.1330). The ultimate load of the SIS complex (5.99 ± 0.37 N) was statistically significant compared with SIS alone (*p* < 0.0001), and also statistically significant compared with PMF and USL (*p* < 0.0001). The ultimate load of the PP complex (10.02 ± 1.80 N) was not statistically significant compared with PP alone (*p* = 0.0846), but was statistically significant compared with PMF and USL (*p* < 0.0001; Fig. 3).

**Table 1:** Ultimate load of native tissues, meshes, and mesh complexes, and collagen ratio of uterosacral ligament (USL) and mesh complexes

Specimen	Ultimate load (N)	*p*	Collagen ratio (%)
PMF	1.25 ± 0.85		None
USL	0.66 ± 0.41		36.66 ± 11.64
PCL	0.66 ± 0.05	0.0120	None
PCL complex	1.71 ± 0.41		48.11 ± 9.88
SIS	14.53 ± 0.86	< 0.0001	None
SIS complex	5.99 ± 0.37		75.73 ± 6.20
PP	8.43 ± 0.40	0.0846	None
PP complex	10.02 ± 1.80		81.16 ± 15.86

*PMF* parametrial fascia, *PCL* polycaprolactone, *SIS* small intestinal submucosa, *PP* polypropylene

### Collagen Ratio of Three Different Mesh Complexes

**Fig. 4:**
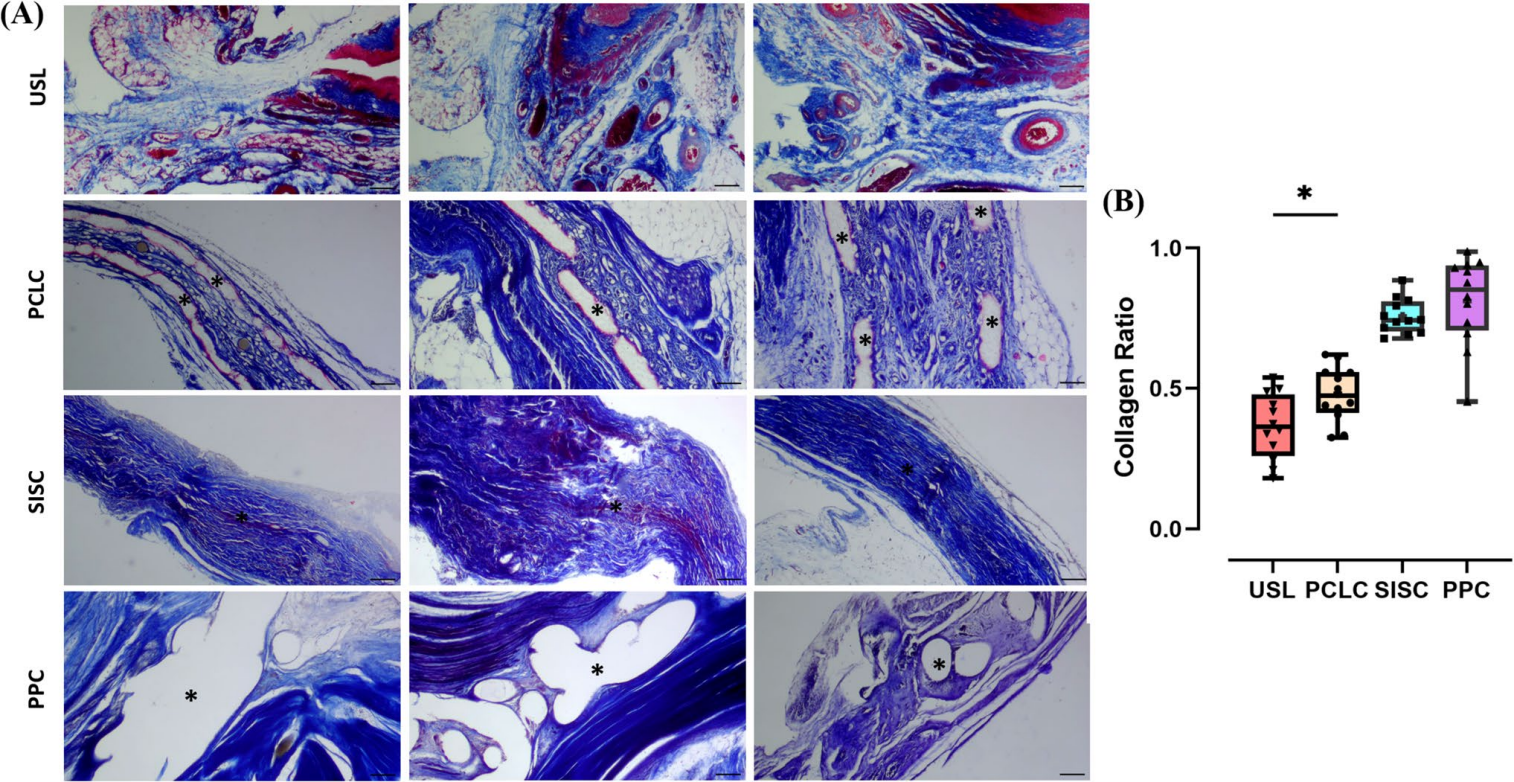
Masson’s Trichrome staining and collagen ratio. **A** The results of Masson staining. Uterosacral ligaments (USLs) are from normal female rats. Polycaprolactone scaffolds complex (PCLC), small intestinal submucosa scaffolds complex (SISC) and polypropylene mesh complex (PPC) are from 4 weeks of unilateral presacral suspension prolapsed rats. Asterisk indicates mesh. Scale bar = 10 μm. **B** Statistical analysis results of collagen ratio. *p < 0.05. Data are presented as mean ± standard deviation

As shown in Table 1 and Fig. 4, the PP complex had the highest average collagen ratio (81.16% ± 15.86%), followed by the SIS complex (75.73% ± 6.20%), whereas the PCL complex had the lowest average collagen ratio (48.11% ± 9.88%). The collagen ratio of the PCL complex was statistically significant compared with the USL (36.66% ± 11.64%; *p* = 0.0164).

## Discussion

This study successfully simulates human pelvic floor repair surgery—sacrocolpopexy—in rodent models. The UPS surgery is brief, taking 42.9 ± 4.5 min, with clear and simple steps, minimal bleeding, easy replication, and rapid postoperative recovery with few complications.

Four weeks after UPS, three different biomaterials formed ligament-like structures with surrounding tissues, referred to as mesh complexes (Fig. 2C, D). Owing to the fragile posterior peritoneum of rats, the PMF and the USL were chosen as comparison objects for the mechanical properties of mesh complexes. There was no significant difference in the ultimate load between PCL and the USL (*p* = 0.9743). Compared with PCL, the ultimate load of the PCL complex increased (*p* = 0.0120), similar to that of the PMF (*p* = 0.4292) and higher than that of the USL (*p* = 0.0204). This increase in the ultimate load of the PCL complex may be related to the increase in collagen content (compared with the USL, *p* = 0.0164). The ultimate load of the PP complex also increased, but not significantly (*p* = 0.0846). This may be because 4 weeks is insufficient to form a stronger structure, and the mechanical properties of PP are much greater than those of the USL and the PMF; thus, the mechanical properties provided by collagen do not make a significant difference.

However, the SIS complex showed the opposite trend. Compared with the USL, the collagen content of the SIS complex increased (*p* < 0.0001), whereas the ultimate load decreased compared with SIS (*p* < 0.0001). Owing to the good biocompatibility of SIS, SIS implants can be gradually degraded in vivo, potentially leading to a decline in mechanical properties. It remains unclear whether the mechanical properties of SIS decline further over time, eventually becoming similar to the supporting tissues of the uterus (like the PMF and the USL).

The main limitation of this study is that it does not fully mimic the process of sacrocolpopexy. Compared with humans, rats have muscle groups in front of the coccyx that control the tailbone. During the UPS, these muscles need to be separated to expose the ligament of the first and second sacral vertebral space. When securing the mesh, it is often fixed together with the ligament and muscles. Even after a 4-week observation period, the rats did not exhibit anxiety, reduced food intake, weight loss, or difficulty urinating. However, the effects over a longer observation period remain unknown.

In this study, we established a UPS in rats to mimic sacrocolpopexy for treating POP. This provides an animal surgery protocol to test different materials in the pelvic region. We used three different types of materials to treat POP in rats. PCL, as an absorbable material, is currently used primarily in clinical settings to promote the repair of skin wounds and it has a high affinity with cells, facilitating cell adhesion [29, 30]. The mechanical properties of PCL are relatively poor, but they improve with collagen formation, making its collagen content closest to that of the USL. If its mechanical properties can be enhanced in the future, it may become a suitable material for pelvic floor repair in large animals (such as pigs, sheep, nonhuman primates, etc.) or humans. SIS, as an acellular biological extracellular matrix, is currently employed in clinical settings for applications including wound repair, cardiac repair, cardiovascular repair, and meniscal repair [31–34]. SIS has strong mechanical properties. Owing to its absorbability, SIS may reduce the incidence of mesh-related complications, but whether its long-term mechanical properties can meet the requirements of the pelvic floor remains uncertain. PP is a stiff, nondegradable material with excellent mechanical properties, commonly used in clinical treatments for POP. However, its complications, such as pain, erosion, and infection, are significant concerns that urgently need to be addressed.

## Conclusion

The UPS successfully simulates human sacrocolpopexy surgery. Zero mortality at 4 weeks postoperation supports the potential for longer-term observation of foreign-body reactions between the mesh and the retroperitoneum. PCL, after reacting with the retroperitoneum, gradually enhances its mechanical properties, and its collagen content is comparable with that of the rat's own tissue. However, the mechanical properties of the mesh are weak, and only by strengthening its mechanical properties can it be applied to larger animals. The SIS mesh inherently possesses excellent mechanical properties and can form dense collagen tissue after reacting with the retroperitoneum. Owing to its absorbable nature, it can avoid complications such as mesh exposure and may potentially replace PP mesh in clinical pelvic floor treatments. This surgery mimics the classical surgery for human POP in rats. First, this procedure can investigate the mechanical properties of pelvic floor tissues at the cellular level after correcting POP. Second, it can be used to validate new materials for surgical treatment of POP, including but not limited to, foreign-body reactions with surrounding tissues, absorption time, etc. Third, it can be used to study the biological mechanisms of mesh erosion.

## Data Availability

The authors could provide the details of results after possible requests.

## Abbreviations

PCL: Polycaprolactone
PMF: Parametrial fascia
POP: Pelvic organ prolapse
PP: Polypropylene
SIS: Small intestinal submucosa
UPS: Unilateral presacral suspension
USL: Uterosacral ligament
